# Integrating nociplastic pain into neuropathic pain framework: a proposal for a revised classification

**DOI:** 10.3389/fpain.2025.1661667

**Published:** 2025-10-30

**Authors:** Katsuhiro Toda

**Affiliations:** Department of Orthopedic Surgery, Yamaguchi Heisei Hospital, Iwakuni, Yamaguchi Prefecture, Japan

**Keywords:** nociplastic pain, neuropathic pain, nociceptive pain, psychogenic pain, central sensitivity syndrome, central sensitization, fibromyalgia, integration

## Abstract

From an etiological perspective, the International Association for the Study of Pain (IASP) classifies pain into three categories: nociceptive pain (NcP), neuropathic pain (NeP), and nociplastic pain (NpP). In clinical practice, distinguishing between NpP and NeP can be particularly challenging. They share many clinical characteristics, including pain hypersensitivity and spontaneous pain. Currently, no evidence-based diagnostic method has been established for NpP, as defined by the IASP. Questionnaires had been developed before the IASP officially adopted NpP in 2017. Therefore, they cannot reliably distinguish between NpP and NeP. There is a previously unrecognized academic ambiguity arising from the coexistence of the unified concept of NeP including NpP and the separate concept of NeP excluding NpP. Moreover, NpP diagnosed using different criteria can coexist. There is currently no established guideline for either the pharmacological or the non-pharmacological treatment of NpP. The treatment of fibromyalgia, a typical NpP, closely resembles that of NeP. The theoretical concept of NpP has generated substantial uncertainty not only in pain research but also in clinical practice, particularly regarding diagnosis and treatment. To simplify diagnosis and treatment, resolve scholarly uncertainty, and improve the care of patients with pain, four provisional plans are proposed until an evidence-based method for diagnosing NpP has been established. (1) Integrate NpP into NeP and use the term NeP. (2) Classify NpP as a subcategory of NeP and use the term NeP. (3) Integrate NpP into NeP and use the term new NeP (nNeP). (4) Classify NpP as a subcategory of NeP and use the term nNeP. The recommended plan is (1). It is hoped that these four proposals will serve as constructive contributions toward advancing both the conceptual understanding of pain and its treatment.

## 1 Introduction

Pain is classified based on various factors, including etiology and duration. From an etiological perspective, the International Association for the Study of Pain (IASP) classifies pain into three categories: nociceptive pain (NcP), neuropathic pain (NeP), and nociplastic pain (NpP). While this classification aims to enhance clinical understanding, the introduction of NpP has introduced significant complexity to both diagnosis and treatment. Furthermore, there is a previously unrecognized academic ambiguity arising from the coexistence of the unified concept of NeP including NpP (unified NeP) and the separate concept of NeP excluding NpP (separate NeP). Moreover, NpP diagnosed using different criteria can coexist. From both diagnostic and therapeutic perspectives, the distinction between NpP and NeP may be of limited clinical relevance. These approaches aim to resolve inconsistencies in pain classification and simplify clinical decision-making, especially for non-specialist clinicians.

To simplify diagnosis and treatment, resolve this scholarly uncertainty, and improve the care of patients with pain, a provisional plan is proposed to integrate NpP into NeP and adopt the term NeP, along with three preliminary proposals. Details are provided in Section 5.

## 2 Methods (narrative review): literature search and selection criteria

Pain management, particularly the pharmacological treatment of chronic pain, is the primary area of specialization. Over the past 25 years, the titles of all PubMed-indexed articles containing keywords such as NSAIDs, acetaminophen, anxiolytics, antidepressants, neuropathic pain, fibromyalgia, and complex regional pain syndrome have been systematically reviewed. Abstracts of articles of interest were examined, and the full texts of the most important papers were obtained and read. Additional keywords included “nociplastic pain,” “pregabalin,” “mirogabalin,” “myofascial pain,” and “trigger points.” These articles were organized into a table-of-contents-style list for easy reference, and this review primarily cites papers from that list. Over the past year, information for this review has also been collected through PubMed keyword searches and AI (ChatGPT). Although the exact keywords used in PubMed searches were not always recorded, the existence of all articles suggested by AI (ChatGPT) was independently verified. This review includes articles indexed in PubMed up to September 11, 2025. This review focuses mainly on papers highlighting problems or confusion caused by NpP, and does not cite papers reporting clinical benefits.

## 3 Current pain classification: background and development

### 3.1 Historical development of pain classification

The IASP formally established the terms NcP and NeP, and their definitions have evolved over time (1). In 1994, NeP was defined as pain initiated or caused by a primary lesion or dysfunction in the nervous system (2), and, in 2011, as pain caused by a lesion or disease of the somatosensory nervous system (3). In 1994, the term NcP did not appear in the book published by the IASP (2). In 2001, NcP was defined as pain that arises from actual or threatened damage to non-neural tissue and is due to the activation of nociceptor (3). In 2016, the IASP first introduced the term NpP (1) and, in 2017, formally adopted it (4).

### 3.2 Current IASP classification: definitions and criteria

Currently, the IASP defines NcP, NeP, and NpP as follows (5).

NcP is pain that arises from actual or threatened damage to non-neural tissue and is due to the activation of nociceptors (5). This term is designed to contrast with NeP (5). The term is used to describe pain occurring with a normally functioning somatosensory nervous system to contrast with the abnormal function seen in NeP (5).

NeP is pain caused by a lesion or disease of the somatosensory nervous system (5). NeP is a clinical description (and not a diagnosis) which requires a demonstrable lesion or a disease that satisfies the established neurological diagnostic criteria (5). The term lesion is commonly used when the diagnostic investigations (e.g., imaging, neurophysiology, biopsies, lab tests) reveal an abnormality or when there was obvious trauma (5). The term disease is commonly used when the underlying etiology of the lesion is known (e.g., stroke, vasculitis, diabetes mellitus, genetic abnormality) (5).

NpP is pain that arises from altered nociception despite no clear evidence of actual or threatened tissue damage causing the activation of peripheral nociceptors or evidence for disease or lesion of the somatosensory system causing the pain (5). Patients can have a combination of NcP and NpP (5).

Several conceptual challenges remain. First, there is currently no evidence-based method to diagnose NpP as defined by the IASP. Second, the distinction between NeP and NpP depends largely on whether lesions can be detected using current diagnostic technologies, which may change in the future. Third, the coexistence of the unified NeP and the separate NeP, both merely labeled as “NeP,” represents a previously unrecognized academic ambiguity. Details are discussed in Sections 4.1 and 4.5.

### 3.3 Representative examples of pain types

Thapa et al. (6) and many others present specific conditions corresponding to NcP, NeP, and NpP. Table 1 lists the specific conditions that are generally recognized as typical conditions of each pain type. It is estimated that the typical conditions of NcP are post-traumatic pain and osteoarthritis, those of NeP are diabetic neuropathy and postherpetic neuralgia (PHN), and that of NpP is fibromyalgia (FM).

**Table 1:** Mechanistic types of pain.

Pain type	Examples
Nociceptive pain	Osteoarthritis, Rheumatoid arthritis, Post-traumatic pain
Neuropathicc pain	Radiculopathy, Diabetic neuropathy, Postherpetic neuralgia
Nociplastic pain	Fibromyalgia, Chronic back pain, Chronic temporomandibular pain disorders

## 4 Limitations of the current classification

### 4.1 Conceptual challenges of nociplastic pain

Kosek et al. emphasized that the proposal to introduce a third pain mechanism should itself be subject to debate (1). This “third mechanism” refers to the concept later proposed as NpP.

The three aforementioned types of pain are defined based on their etiology. NcP can be generally identified based on pain etiology. According to the IASP definition, NeP is diagnosed when a lesion or disease of the somatosensory nervous system accounts for the pain in cases not classified as NcP. Among pain that is not NcP, pain lacking such a lesion or disease is classified as NpP. Specifically, the level of medical knowledge and diagnostic technology at the time of diagnosis determines whether a lesion or disease can be identified to explain the etiology of pain. Whether such a lesion or disease is identified or not distinguishes NeP from NpP. Caution is warranted when relying on this method of classification.

For example, in ancient times, no lesions could be identified in Parkinson's disease. Ancient medical theories (e.g., demonic possession or ancestral curses) based on the inability to identify lesions have been rejected by current medical evidence. Lesions in Parkinson's disease have been identified using current advanced medical technologies. Similarly, any medical theories based on the inability to detect lesions with current technology—which is far less advanced than what may be available 100 years from now—may also become obsolete.

FM is generally considered a typical NpP. At present, no cerebral abnormalities suitable as diagnostic criteria have been identified; however, various cerebral abnormalities have been reported (7, 8). Many of those studies have been conducted using research-grade brain examination methods rather than those commonly used in clinical practice. Due to variability in the locations of abnormalities across studies, the specific brain region responsible for FM has not been determined. In the future, advancements in diagnostic technologies (including diagnostic equipment) may enable the detection of lesions that explain the etiology of pain. Therefore, FM may eventually be reclassified from NpP to NeP. Such a retransition could result in conceptual inconsistencies and contradictions.

NeP is typically divided into central NeP, arising from lesions in the central nervous system (CNS), and peripheral NeP, which arises from lesions in the peripheral nervous system. Traditionally, most cases of NeP have been considered peripheral NeP, whereas conditions currently described as NpP may share some features with central NeP.

### 4.2 Diagnostic challenges in pain

#### 4.2.1 Neuropathic pain and nociplastic pain: diagnostic methods and limitations

Currently, four primary methods are used to diagnose NeP and NpP: the IASP definition, the grading system, questionnaires, and objective diagnostic methods. However, currently, no evidence-based diagnostic method has been established for NpP, as defined by the IASP.

##### 4.2.1.1 Distinguishing nociplastic pain and neuropathic pain based on IASP definitions

In cases of pain not classified as NcP, the presence of disease or lesion that explains the pain indicates NeP, whereas its absence indicates NpP, according to the IASP definition. However, the readers of the definition have to decide for themselves what is clear and what is unclear evidence of disease or lesion (9).

Abnormalities in brain or spinal cord CT or MRI have been proposed as potential biomarkers for NeP. Post-stroke pain, classified as NeP, is typically associated with lesions detectable on CT or MRI. However, these imaging modalities cannot consistently distinguish between painful and painless stroke. In clinical practice, the patient's subjective report—such as the presence or absence of pain—remains a key component in diagnosing painful stroke. Given the difficulty in distinguishing stroke lesions that cause pain from those that do not, classifying NeP and NpP solely based on the presence or absence of a detectable lesion or disease may be unreliable.

At present, no diagnostic technologies exist that can reliably distinguish stroke with pain from stroke without pain, without relying on the patient's self-reported pain. Consequently, the strict differentiation of NeP from NpP based solely on objective lesion evidence is limited and may lead to diagnostic failure. At present, in other words, although the IASP definitions of NeP and NpP are theoretically useful, their clinical applicability remains limited. This diagnostic discrepancy may further complicate patient selection in clinical trials and impede the evaluation of the efficacy of new therapies.

##### 4.2.1.2 Challenges with grading system

Treede et al. introduced the grading system in 2008 (10), followed by Haanpaa et al. in 2011 (11), Finnerup et al. in 2016 (12), and Kosek et al. in 2021 (13). The grading systems reported by Treede et al., Haanpaa et al., and Finnerup et al. serve as the diagnostic criteria for NeP (10–12), while the grading system proposed by Kosek et al. serves as the diagnostic criteria for NpP (13).

A draft of Treede et al.'s paper was reviewed by the NeuPSIG (IASP Special Interest Group on Neuropathic Pain) management committee prior to submission (10). The work of Haanpaa et al. was partially supported by travel grants from NeuPSIG, the Special Interest Group on NeP of the IASP (11). The study by Finnerup et al. was supported by the NeuPSIG of the IASP (12). The study by Kosek et al. was primarily established by the IASP Terminology Task Force (13). In short, the grading systems were essentially established by the IASP.

The grading system for diagnosing NpP was created by a consensus of pain experts (13). Kosek et al. reported: Currently, NpP is graded as “possible” or “probable,” but not “definite” (13). If future diagnostic tests are developed and validated, the introduction of the term “definite NpP” should be considered (13). Currently, no widely validated diagnostic criteria exist for “definite NpP.” There is no evidence that NpP diagnosed using the grading system is consistent with NpP as defined by the IASP.

Definite NeP in the grading system must meet the following conditions: Negative or positive sensory signs, confined to the innervation territory of the lesioned nervous structure (11). However, Lynch et al. reported as follows: The requirement that positive sensory findings (allodynia, hyperesthesia, hyperalgesia) be “confined to the innervation territory of the lesioned neural structure” has not been validated; furthermore, it is inconsistent with the current evidence (14). Schmidt et al. reported that the low sensitivity (60%) suggested that the published grading system (13) was not suitable for screening purposes (15). They suggested structural and content modifications to improve sensitivity (15). Hoegh et al. reported that the proposed grading system has specific problems, including low specificity and sensitivity, and that the NpP concept itself presents issues such as unclear definitions and overlaps with other pain mechanisms (16).

Bilika et al. conducted a reliability and diagnostic accuracy study using 32 clinical vignettes derived from previously published cases and existing clinical data (17). They reported as follows: The validity of the criteria was reported to be moderate (*κ* = 0.68), with strong specificity (89.0%) and moderate sensitivity (69.0%) (17). Positive and negative predictive values were also high, at 81.8% and 81.0%, respectively, supporting the proposed criteria's accuracy in identifying and excluding NpP (17). NeP classification is inconsistent, with some cases including NpP and others excluding it. Clinical vignettes constructed from such inconsistent data may therefore reflect imprecise or unstable criteria. When diagnostic criteria are clearly defined, statistical indicators such as specificity, sensitivity, and predictive values are highly valuable. When a correct diagnosis cannot be made, these indicators may become unreliable.

##### 4.2.1.3 Distinguishing nociplastic pain and neuropathic pain based on questionnaires

Questionnaires such as the Leeds Assessment of Neuropathic Symptoms and Signs (LANSS) Pain Scale (18), the self-report version of LANSS (S-LANSS) (19), the painDETECT questionnaire (PD-Q) (20), the Douleur Neuropathique 4 Questions (DN4) (21), the self-completed DN4 (S-DN4, the modified version of DN4) (22), and the ID Pain (23) have been developed to identify NeP. These questionnaires were initially developed using data on patients with predominantly NcP and those with predominantly NeP. If the score exceeds a certain threshold, the pain is categorized as being likely NeP. Notably, osteoarthritis was classified as predominantly NcP during the development of these questionnaires, although this classification has recently been questioned. Prolonged pain can sensitize the CNS, causing pain (24–28). Illeez et al. reported that osteoarthritis pain, which is a complex outcome with nociceptive and neuropathic components, led to central sensitization (CS) in a chronic phase (29). Antidepressants, which are medications for the treatment of NeP, are often effective in osteoarthritis (30). Knee osteoarthritis was considered to be predominantly NcP when the questionnaires were first developed. However, when patients with knee osteoarthritis were assessed with the questionnaires, the prevalence of NeP ranged from 13.65% (31), 23% (32), 20%–40% (33), to almost half (34). Therefore, these questionnaires fail to reliably distinguish between patients with pure NcP and pure NeP. This raises critical questions regarding which specific types of pain these questionnaires can truly identify with validity and accuracy. A letter to the editor posed this question to the developer of the PD-Q (20, 27); however, no response has been provided (35).　

Mayer et al. reported the Central Sensitization Inventory (CSI) in 2012 (36). Scores of 40 and above were considered indicative of CS (37). It was a self-report screening instrument to evaluate CS-related clinical symptoms in chronic pain populations (36) and to help identify patients with central sensitivity syndrome (CSS), including FM (37).

Most questionnaires, as well as objective diagnostic methods, had been developed before the IASP officially adopted NpP in 2017. High scores that make the diagnosis of NeP with the questionnaires are more likely to be observed in cases of NpP or secondary central sensitization process (38). Therefore, they cannot reliably distinguish between NpP and NeP. For more details, see Section 4.5 and 4.6.

##### 4.2.1.4 Objective diagnostic methods

Various diagnostic tests, including quantitative sensory testing, neurophysiology, skin biopsy, corneal confocal microscopy, functional neuroimaging, genetic testing, and other related methods, have been reported as diagnostic tools to identify NeP (39). These tests evaluate different physiological parameters, yet they provide limited utility in distinguishing NpP from NeP. Imaging and blood tests are included in objective diagnostic methods. Although several potential biomarkers have been proposed, none have been formally validated for NpP and NeP using imaging or blood tests. Biomarkers that distinguish NpP from NeP do not exist either.

#### 4.2.2 Difficulty distinguishing nociplastic pain and neuropathic pain

##### 4.2.2.1 Diversity and limitations of diagnostic approaches

The four approaches to NeP—namely, the IASP definition, the grading system, questionnaires, and objective diagnostic methods—do not necessarily identify the same set of clinical entities. In clinical practice, distinguishing between NpP and NeP can be particularly challenging. They share many clinical characteristics, including hypersensitivity to pain, spontaneous pain, and overlapping treatments considered effective for both conditions. Medical theory, including the IASP definition of the three types of pain, may not align with the diagnostic methods employed in clinical practice. In particular, standardized diagnostic criteria for NpP are still under development, and there are currently no widely accepted tests that can reliably distinguish NpP from NeP in everyday clinical practice.

NpP has many overlapping mechanisms and clinical features with NeP, potentially leading to terminological confusion and misclassification (9). Hoegh et al. reported that the proposed grading system has specific problems, including low specificity and sensitivity, and that the NpP concept itself presents issues such as unclear definitions and overlap with other pain mechanisms (16).

##### 4.2.2.2 Fibromyalgia as an example: overlap between nociplastic pain and neuropathic pain

FM is a prototypical form of NpP (6, 9). However, FM is closely associated with NeP.

Historically, before the formal adoption of NpP, FM had been reported as NeP (40, 41). FM is reported to be included in CSS (42–47). Martínez-Lavin reported as follows: FM can be conceptualized as an NeP syndrome and there is no need to propose NpP as new chronic pain mechanism (48).

Many recent studies have found that FM is frequently associated with small fiber neuropathy (49–57). It reinforces the dysautonomia-neuropathic hypothesis and validates FM pain (50). FM, a prototypical form of NpP, is often classified as NeP based on questionnaire results (56, 58, 59). Findings from painDETECT and LANSS suggest that FM may have a neuropathic pain component (60).

Marshall et al. reported that pain phenomenology in patients with FM syndrome showed considerable overlap with NeP (61). It has been reported that patients with FM are often classified as NeP based on the results of PD-Q and DN4 (58). Galiero et al. reported that 9 of 22 FM patients were DN4 positive (NeP) (59). Viceconti et al. reported that 62.4% of FM patients had Neuropathic Pain Symptom Inventory (NPSI) scores of ≥50/100, suggesting severe NeP (56). Elsawy et al. concluded that moderate to severe NeP was experienced by all the studied patients with FM syndrome, and small fiber pathology was suggested to be a significant contributor to NeP (62). Thus, at least half of patients with FM—a condition typically classified as NpP—are identified as having NeP based on questionnaire results.

##### 4.2.2.3 Limitations of questionnaires as screening tools

Despite using all four tests (S-DN4, the ID Pain, PD-Q, and S-LANSS), between 20% and 30% of patients with an NeP component were missed (63). Gudala et al. stated as follows: Thus, these questionnaires can only be used as an initial clue in screening for NeP components, but do not replace clinical judgment (63).

When PD-Q, S-LANSS, and DN4 were used to diagnose NeP in patients with polyneuropathy, the probability of obtaining a correct test result was three-quarters at best, and at worst, no better than two-fifths (64). Dunker et al. reported as follows: Therefore, neither DN4, PD-Q, nor S-LANSS can be confidently used to assess NeP in a neurological outpatient population with symptoms of polyneuropathy (64).

##### 4.2.2.4 Limitations in pediatric and adolescent assessment

A systematic review of psychometric properties and feasibility has identified methods to discriminate between NcP, NeP, and NpP in children and adolescents (65). This systematic review highlights the limited validation of pain assessment tools for children and adolescents (65). While quantitative sensory testing and self-reported questionnaires show promise in identifying pain mechanisms, their applicability remains uncertain (65).

#### 4.2.3 Differences between basic science and clinical practice

It has been proposed that NpP and NeP are mechanism-based descriptors of pain rather than formal diagnostic categories or distinct disease entities, potentially creating a disconnect between basic science and clinical practice. Mechanism-based descriptors without established diagnostic methods or specific treatments have limited clinical relevance.

NcP and NeP are defined by the IASP based on pain mechanisms, and from the mechanistic perspective, they can be diagnosed to some extent, with specific treatment strategies available for each. In this case, the gap between basic science and clinical practice is small. However, currently, there is no established method to specifically diagnose NpP based on the IASP-defined pain mechanisms, nor are there treatments specifically targeting NpP. Consequently, the gap between basic science and clinical practice is large for NpP. One potential solution is the integration of NpP into NeP.

Another possible approach is to develop methods that can specifically diagnose NpP based on the IASP definition and identify specific treatments. When conducting interventional studies for NpP, the lack of established diagnostic criteria for NpP that exclude NeP makes patient selection challenging. Simply testing treatments effective for NeP has limited clinical significance; under the current circumstances, applying diagnostic and treatment strategies established for NeP is reasonable. Conducting interventional studies for NpP risks fragmenting the evidence base for NeP, potentially adversely affecting patient care and the development of novel therapies.

An intervention study of At Home Morning Bright Light Treatment for NpP is currently being conducted (66). In this study, patients meeting the diagnostic criteria for FM were classified as NpP. Photobiomodulation therapy (PBM) has been reported to be effective for diabetic peripheral neuropathy (67, 68), FM (69–72), osteoarthritis, and various other conditions (73, 74). Therefore, At Home Morning Bright Light Treatment may be beneficial for patients with FM. Although the ongoing study (66) is well-designed as an intervention study for FM, as noted in Section 4.2.2.2, FM represents a mixed pain condition involving both NeP and NpP. Accordingly, it is not appropriate to evaluate this study as an intervention study for NpP. This issue may cause confusion both in clinical practice and in basic science. Another interventional study did not specify the diagnostic criteria for NpP and enrolled “patients with mixed type (neuropathic and nosiplastic pain phenotype)” (75). Therefore, it is also not appropriate to evaluate this study as an intervention study for NpP. Because there is currently no reliable method to accurately identify NpP in the absence of NeP, caution is required when interpreting studies targeting NpP.

Nijs et al. reported as follows: the BACPAP consortium's consensus recommendations—namely, that low back pain is characterized by dominant nociceptive, neuropathic, or nociplastic mechanisms, and that personalized pain medicine is based on pain phenotypes—are not ready to be implemented in clinical practice until additional evidence is generated that is specific to these low back pain phenotypes (76).

### 4.3 Therapeutic challenges

Because NcP and NeP require markedly different treatments, distinguishing between the two types of pain is essential from both diagnostic and therapeutic perspectives. Pharmacological treatments for NcP include non-steroidal anti-inflammatory drugs (NSAIDs) and acetaminophen, whereas NeP is typically managed with serotonin–norepinephrine reuptake inhibitors (SNRIs), amitriptyline, and gabapentinoids (77). There is currently no established guideline for either the pharmacological or the non-pharmacological treatment of NpP. The treatment of FM, a typical NpP, closely resembles that of NeP. Kosek reported that the pharmacological treatment of NpP closely resembles that of NeP but differed from NcP (78).

Several medications have demonstrated efficacy in treating NeP associated with diabetic neuropathy and PHN, and these medications are also often used to treat NeP associated with other conditions (79). While the pharmacological and non-pharmacological treatments for diabetic neuropathy, PHN, post-stroke pain, spinal cord injury pain, and other NeP conditions differ in certain respects, the overall treatment strategies are largely similar. Likewise, while small distinctions exist between the treatment strategies for FM (a typical NpP) and various forms of NeP, from a practical perspective, they may be considered largely interchangeable. For clinicians specializing in pain treatment, especially those focusing on the pharmacological treatment of chronic pain, memorizing specific treatments for each pain subtype can be considered somewhat impractical. For physicians who are not pain specialists, remembering the distinctions between treatment strategies may be even more challenging. Antidepressants (SNRIs and amitriptyline) and gabapentinoids have shown efficacy in both NeP and FM (80, 81). Similarly, the non-pharmacological treatments such as exercise (82, 83), cognitive behavioral therapy (CBT) (84, 85), transcranial direct current stimulation (tDCS) (86, 87), and mindfulness-based interventions (88, 89) have shown efficacy in both NeP and FM. In clinical practice, treatments effective for FM have also been applied to NeP, and vice versa. This cross-application has broadened treatment strategies and may have contributed to improved clinical outcomes in both conditions. Placing emphasis on the subtle differences between treatments for NpP (or FM) and NeP is questionable in terms of clinical value, as the treatment strategies are largely similar, if not virtually identical. Because the treatments for NpP (or FM) and NeP are very similar, the clinical value of distinguishing between them is questionable.

### 4.4 Challenges in clinical practice

**Figure 1:**
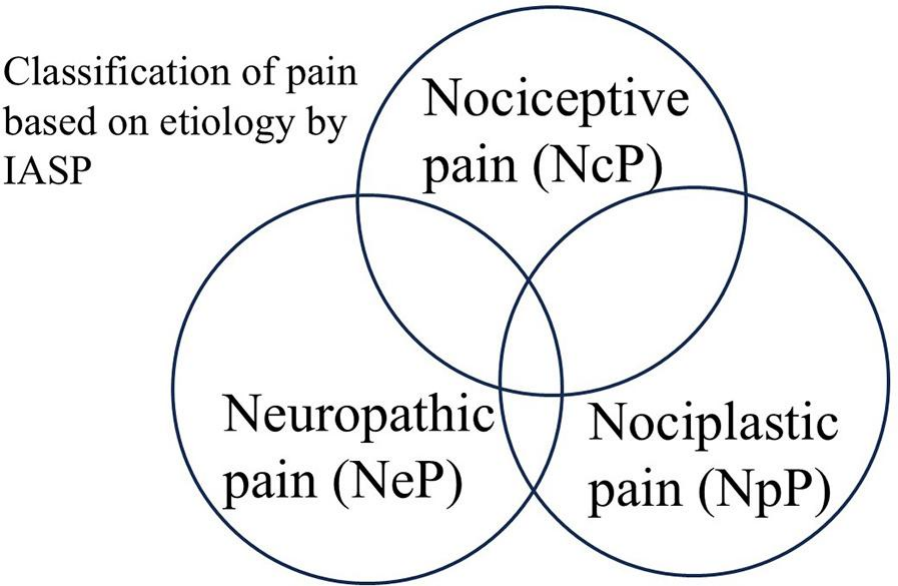
Classification of pain based on etiology by IASP. This figure illustrates the potential overlaps among nociceptive pain (NcP), neuropathic pain (NeP), and nociplastic pain (NpP). The intersections represent pain conditions involving multiple mechanisms—nociceptive, neuropathic, and nociplastic—highlighting the complexity of pain classification and diagnostic challenges in clinical practice.

The conceptual framework that emphasizes minor distinctions between treatments for NpP and NeP may complicate the overall approach to pain treatment. Pain is classified into seven categories (NpP + NeP + NcP, NpP + NeP, NpP + NcP, NeP + NcP, NpP, NeP, and NcP) based on the combinations of the three types of pain (see Figure 1). Even among clinicians specializing in pain treatment, particularly in the pharmacological treatment of chronic pain, making distinctions among these seven categories in clinical practice can be challenging. Moreover, clearly distinguished treatment strategies for each category have not been well established. From a treatment perspective, a clear distinction between NpP and NeP is often unnecessary. Non-specialist clinicians are likely to find pain diagnosis and treatment too complex, making it difficult for them to provide appropriate care. From both diagnostic and therapeutic perspectives, the distinction between NpP and NeP may be of limited clinical relevance.

### 4.5 Challenges from an academic perspective

Most questionnaires, as well as objective diagnostic methods, were developed before the IASP officially adopted NpP in 2017. Therefore, they cannot reliably distinguish between NpP and NeP. This challenge is particularly pronounced for questionnaires, which are used more frequently. Because NpP shares many clinical features with NeP, it is likely that some cases of NpP are classified as NeP in the context of these questionnaires. This challenge is discussed in greater detail later. As discussed later, FM, a typical example of NpP, is often classified as NeP by these questionnaires.

Therefore, caution is warranted when interpreting the results of these questionnaires as evidence of NeP in such contexts.

There are two conceptualizations of NeP: the unified NeP and the separate NeP. Currently, the same term NeP is used with different meanings. The term NeP in papers published before 2017 likely included the current NpP (i.e., the unified NeP). Therefore, the term NeP in papers published before 2017 thus differs from the current definition of NeP (i.e., the separate NeP). Caution should be exercised when interpreting the term NeP in papers published after 2017. If both NeP and NpP are mentioned in the same paper (90), the term NeP is generally the separate NeP. However, if only NeP is used in a paper, it is often difficult to determine whether the term NeP is the unified NeP or the separate NeP. As noted above, NeP in articles that use questionnaires to identify NeP is highly likely to represent the unified NeP (91). As a result, evidence regarding the prevalence, diagnosis, and treatment of NeP may be developed based on different conceptual frameworks, causing difficulties in accurate interpretation and comparison among studies. This confusion tends to be further exacerbated in narrative reviews, systematic reviews, and meta-analyses citing original papers. Similarly, the confusion tends to gradually worsen over time. This confusion is difficult to detect, and it is extremely challenging for non-specialist clinicians to recognize it. To the best of the author's knowledge, no published study has explicitly described the conceptual uncertainty arising from the simultaneous use of the unified NeP and the separate NeP. The most serious concern is that this conceptual uncertainty may go unrecognized, resulting in the continued accumulation of erroneous evidence and the entrenchment of an erroneous foundation for clinical medicine and basic research.

### 4.6 The central sensitization inventory as one of the substantial diagnostic criteria for nociplastic pain

In this Section 4.6, a CSI score of 40 or higher is used as a diagnostic criterion for CS or CSS.

Iwasaki et al. reported that there was an association between NeP and CSS in patients with preoperative lumbar spinal stenosis using the painDETECT and CSI questionnaires (92). Feng et al. reported that CS was commonly seen in chronic pain disorders, including NeP (93). Mesci et al. reported that significantly higher NeP scores were found in patients with CS compared to those without CS (94). Thus, NeP and CS (or CSS) are closely associated.

Alp et al. reported that among 114 patients with psoriatic arthritis, 16 (66.7%) of the 24 patients with NeP also had CS, while 18 (36.7%) of the 49 patients with CS also had NeP (95). Kim et al. reported that among 316 patients who underwent primary unilateral total knee arthroplasty for end-stage osteoarthritis, 55 (17.4%) had both CS and NeP, 68 (21.5%) had CS only, 35 (11.1%) had NeP only, and 158 (50.0%) had neither condition (96). Among the 103 patients with CS (CSI ≥40), 68 (66.0%) also had NeP. Goldoni et al. reported that among 125 participants with knee osteoarthritis, 33 (26.4%) had both neuropathic-like symptoms (NS) and CS, 18 (14.4%) had NS only, 27 (21.6%) had CS only, and 47 (37.6%) had neither condition with NS assessed using the same diagnostic criteria as NeP (97). Among the 60 patients with CS (CSI ≥40), 33 (55.0%) also had NeP.

Leerling et al. reported that among 80 adult patients with chronic nonbacterial osteitis (CNO), 23 patients (29%) had pure NcP (NcP only, no NeP or NpP components), 2 patients (3%) had a combination of NcP and NeP, 32 patients (40%) had a combination of NcP and NpP, and 23 patients (29%) had a combination of NcP, NeP, and NpP (98). All patients with CNO naturally had NcP. Among the 55 patients with CS (CSI ≥40), 23 (41.8%) also had NeP. They used a CSI score ≥40 as one of the diagnostic criteria for NpP. Their diagnostic criteria for NpP differ from the conventional ones. In other words, NpP diagnosed using different criteria can coexist.

## 5 Proposed reconceptualization of pain classification: integrating nociplastic pain into neuropathic pain

Several authors have expressed skepticism about the necessity of introducing NpP as a separate pain category. Martínez-Lavin stated: “There is no need to propose NpP as new chronic pain mechanism.” (48) Lars-Petter Granan stated: “We do not need a third mechanistic descriptor for chronic pain states! Not yet.” (99)

**Figure 2:**
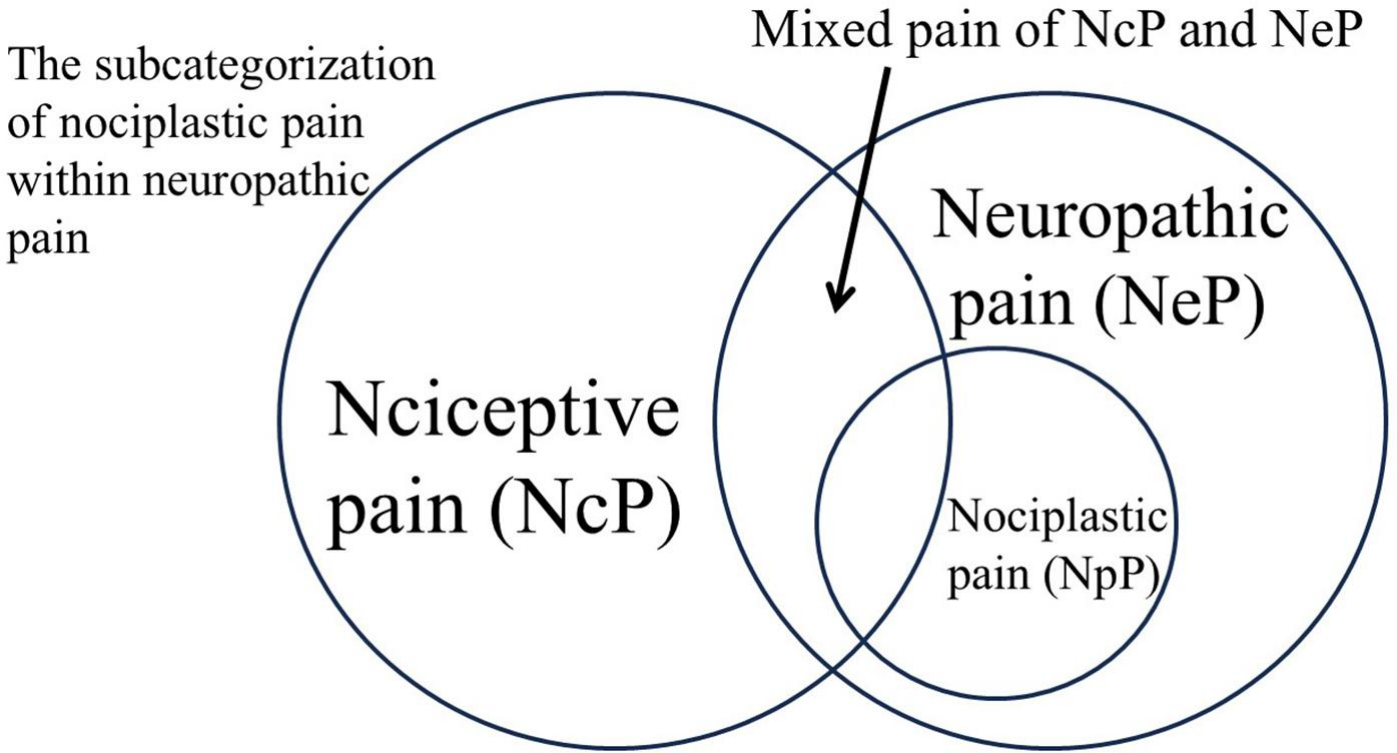
The subcategorization of nociplastic pain within neuropathic pain. This figure illustrates a proposed conceptual model in which nociplastic pain (NpP) is positioned as a subcategory of neuropathic pain (NeP). The outer circle represents the broad category of NeP, encompassing both traditional neuropathic conditions and those formerly classified as NpP. Within this framework, NpP is positioned entirely within NeP, reflecting the growing recognition of shared pathophysiological mechanisms—particularly central sensitization and dysregulated pain modulation.

Currently, the theoretical concept of NpP has generated substantial uncertainty not only in pain research but also in clinical practice, particularly regarding diagnosis and treatment. Until diagnostic methods that can clearly distinguish NeP from NpP are established, new questionnaires that reliably differentiate the two are developed, and differences in their respective treatments are clearly demonstrated, it is proposed to provisionally integrate NpP into NeP and adopt NeP as the provisional unified term. As an alternative approach, the classification of NpP as a subcategory of NeP is also provisionally proposed (see Figure 2). However, the principal proposal of this paper is the integration of NpP into NeP, while the classification of NpP as a subcategory is regarded as a subordinate provisional option.

If the above conditions are eventually met, the concept of NpP could provide considerable benefits for both physicians and patients.

Such integration would necessitate a revised definition of NeP. One possible candidate for redefinition is the 1994 definition of NeP: Pain initiated or caused by a primary lesion or dysfunction in the nervous system (2).

These two proposals may help resolve the substantial uncertainty arising from the simultaneous existence of the separate NeP and the unified NeP from 2017 to the present, extending into the future. Historically, three phases of NeP can be distinguished: (i) NeP before the adoption of the NpP concept, (ii) NeP coexisting with and without NpP, and (iii) the new NeP with NpP internally integrated.

To clarify the coexistence period of the separate NeP and the unified NeP, and to distinguish the new NeP with NpP internally integrated from conventional NeP, it is also proposed to provisionally designate the new NeP as new NeP (nNeP). With this proposal, four possible approaches are presented in this paper:

1. Integrate NpP into NeP and use the term NeP.
2. Classify NpP as a subcategory of NeP and use the term NeP.
3. Integrate NpP into NeP and use the term nNeP.
4. Classify NpP as a subcategory of NeP and use the term nNeP.

The recommended approach is approach 1: integrate NpP into NeP and use the term NeP.

Whether using NeP or adopting nNeP, avoiding the term NpP could help resolve uncertainty in both pain research and clinical practice.

## 6 Potential benefits of the revised framework

### 6.1 Advantages in clinical practice

While only specialists need to understand leukemia treatment, all clinicians should understand pain treatment. Nearly all clinicians encounter and manage pain in their routine practice. Therefore, simplifying the diagnostic framework and treatment strategies—such as integrating NpP into NeP—could improve non-specialist clinicians' ability to provide more effective care.

**Figure 3:**
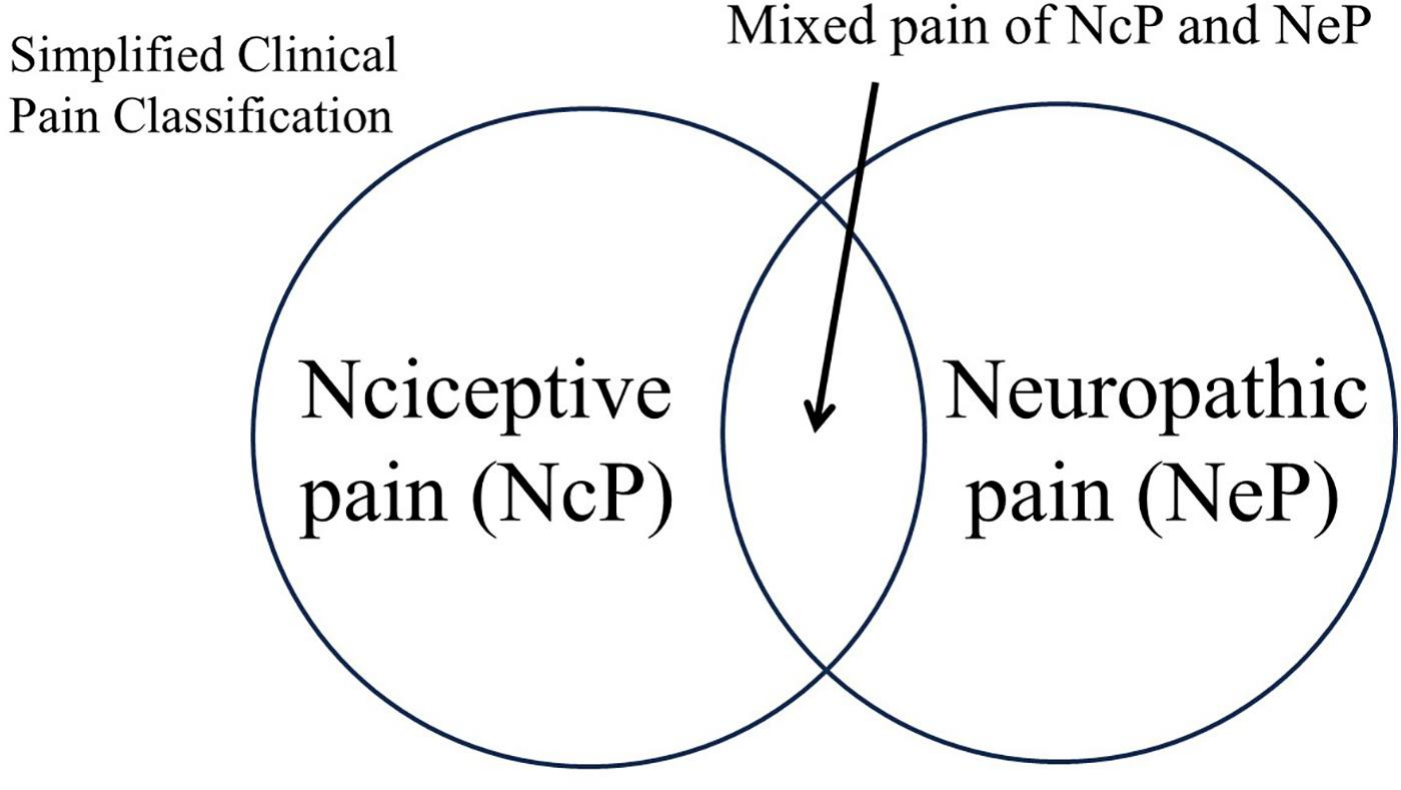
Simplified clinical pain classification. This figure illustrates a simplified classification model for the clinical management of pain. Pain is broadly categorized into nociceptive pain (NcP) and neuropathic pain (NeP), with nociplastic pain (NpP) integrated into NeP. In practice, due to the frequent mechanistic overlap, three clinical categories are proposed: pure NcP, pure NeP (including former NpP), and mixed pain involving both NcP and NeP. This simplified framework is designed to facilitate clearer diagnosis and treatment planning, especially for non-specialist clinicians.

Integrating NpP into NeP may streamline the pain classification framework. In this model, pain would be categorized into two primary types: NcP and NeP. Practically, because mixed pain states frequently occur, pain may be classified into three clinical categories: pure NcP, pure NeP, and mixed pain involving both NcP and NeP (see Figure 3). This simplified classification may facilitate both diagnosis and treatment planning.

Moreover, by unifying NpP with NeP, evidence related to treatment and epidemiology for each can be shared and applied across both conditions, potentially expanding treatment strategies. Broader treatment strategies may result in better treatment outcomes. As described in Section 4.3 Therapeutic Challenges, treatment for non-NcP pain is generally similar. These approaches are also applied to pain with an unknown diagnosis. Exceptions include the pharmacological treatment of trigeminal neuralgia (100), migraine [symptomatic medications (101) and preventive medications (102)], complex regional pain syndrome (103), and others. If exceptional diseases are excluded, there are three possible approaches: NcP treatment, NeP treatment, or a combination of the two. From both diagnostic and therapeutic perspectives, the distinction between NpP and NeP may be of limited clinical relevance.

When differing medical theories exist, it is reasonable to evaluate their utility from a therapeutic outcome perspective. Generally, a greater variety of treatment options can provide more opportunities for individualized and effective care. If treatment strategies traditionally associated with both NeP and NpP are applied within a unified framework, treatment options may be broadened, potentially contributing to better treatment outcomes.

The proposed integration of NpP into NeP also has another important implication. The term “psychogenic pain” is still occasionally used in papers (104–107), and in some cases, is used almost synonymously with NpP (107). For example, one recent study suggests that the CSI score is now thought to assess psychogenic pain (107). It is likely that most forms of pain, including NcP, are influenced by psychological states; however, this influence is distinct from the concept of psychogenic pain. According to the IASP definition, psychogenic elements are not included in the concept of NpP (5). Although the use of the term psychogenic pain appears to be gradually declining, it may still be more commonly encountered in actual clinical practice than in peer-reviewed papers, as it is often altered during the editorial review. Based on clinical experience in Japan, the term “psychogenic pain” is occasionally used, and such a diagnosis is sometimes made (104, 107). In particular, patients labeled as having psychogenic pain are often subjected to psychosocial burden and may be deprived of opportunities to receive appropriate care, raising serious concerns. The proposed reclassification may help reduce the use of this term, ultimately resulting in improved patient care.

In addition to clinical benefits, integrating NpP into NeP may also provide benefits for the scientific community. At present, basic research on NpP and NeP is often conducted separately, resulting in fragmented data and difficulties in comparing findings. In particular, the distinction between CS and NpP remains a major unresolved issue. A unified terminology would facilitate the integration of basic research and promote closer alignment with clinical diagnostic frameworks. Such consistency could accelerate the translation of basic science into clinical applications, ultimately benefiting both research progress and patient care.

### 6.2 Future directions

**Figure 4:**
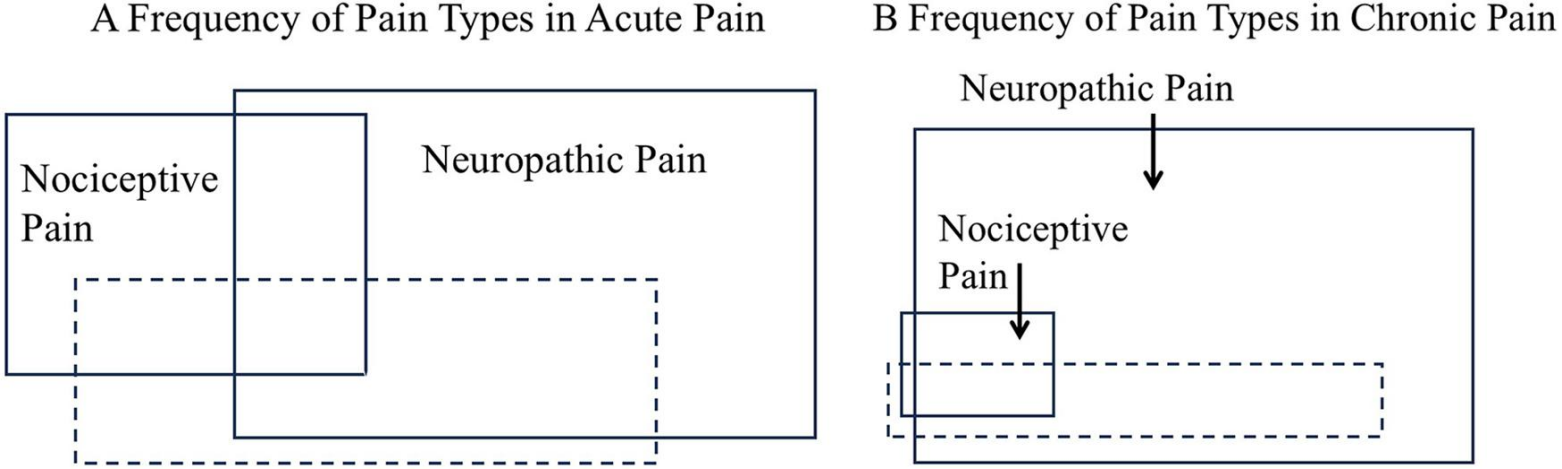
Frequency of different pain types in acute pain **(A)** and chronic pain **(B)**. In acute pain, defined as pain lasting less than three months, pure nociceptive pain (NcP) is relatively common. However, in chronic pain, defined as pain lasting three months or longer, repeated painful stimuli activate the central nervous system, resulting in a reduction in NcP and rendering pure NcP rare. Furthermore, NcP decreases with treatment and over the natural course of the condition. Taken together, these factors further reduce NcP and render pure NcP rare. If neuropathic pain (NeP) is divided into central and peripheral NeP, the figure represents NeP with central NeP replacing it, and the dashed line indicating peripheral NeP. The area indicates the relative frequency of pain types; however, no supporting data exists, and this representation is based on the author's subjective assessment.

Historically, pain research has focused on NcP, whereas NeP research focused on peripheral mechanisms. Because pure NcP is rare and NcP–NeP mixed pain and pure NeP are increasingly relevant, integrating NpP into NeP will likely focus future research on NeP, particularly central NeP. In this framework, FM may be recognized as a prototypical central NeP, due to its high prevalence, including incomplete forms, affecting over 20% of the population (108, 109). In acute pain (<3 months), pure NcP is present in a small proportion of cases (see Figure 4A). In chronic pain (≥3 months), it is nearly absent or may disappear entirely (see Figure 4B).

## 7 Dealing with critical feedback on this study

The problems associated with the theory that categorizes pain as NcP in the absence of an identifiable disease or lesion have already been discussed. Some argue that residual pain, even after clinical remission in rheumatoid arthritis, does not meet the criteria for either NeP or NcP and should therefore be categorized as NpP. However, the residual pain may be more appropriate to classify this pain as a subcategory of NeP. The persistence of pain following remission likely reflects ongoing changes within the nervous system, a hallmark of neuropathic mechanisms rather than that of a separate nociplastic process.

Given this background, integrating NpP into NeP may indeed cause some degree of uncertainty. However, a major source of uncertainty already exists in clinical practice and basic science, where the unified NeP and the separate NeP are frequently used interchangeably without clear distinction. Moreover, a more serious issue is failing to acknowledge this difficulty. Such difficulty is far more substantial than the initial difficulty caused by integration and should be regarded as a challenge to be addressed through integration itself.

As an alternative approach, subcategorizing NpP within NeP is proposed. Compared with the potential uncertainty caused by complete integration, this subcategorization may result in less overall uncertainty.

A potential criticism is that this study includes fundamental errors. There is a view that “NpP already serves as an evidence-based explanation that can help make sense of pain without any known pathology.” (110). However, in practice, NpP is very similar to CS and unexplained cases of central NeP. Although these three terms are defined from different perspectives, they essentially refer to almost the same concept. CS and unexplained cases of central NeP can likewise help make sense of pain without any known pathology. This paper provides a response to other studies suggesting the clinical utility of NpP (see Table 2) (13, 110–113)).

**Table 2:** Selected studies on the claimed utility of nociplastic pain and corresponding responses.

Author (Ref.)	Claim	Response
Kosek (13)	NpP helps patients make sense of their pain and understand what can be done (and what they can do), including the implications for treatment and prognosis.	Because NpP is difficult for non-specialist clinicians to comprehend, patients are likely to find it even harder to understand. Moreover, its diagnostic and therapeutic utility remains limited, as noted in the response to Ablin.
Fitzcharles (111)	It is important to recognise NpP, since it will respond to different therapies than NcP, with a decreased responsiveness to anti-inflammatory drugs and opioids, surgery, or injections.	If NpP is integrated into NeP, the differences in treatment are reduced to those between NeP and NcP, and the distinctiveness and clinical value of NpP are therefore limited.
Bułdys (112)	It is important that physicians widely discuss and acknowledge NpP to provide optimized pain control for patients.	As noted in the response to Ablin, no concrete diagnostic or therapeutic utility has been demonstrated.
Ablin (113)	The recognition and management of NpP are crucial for advancing the care of patients with chronic pain.	There is currently no established diagnostic method that can reliably distinguish NpP from NeP, nor has any treatment been reported to be specifically effective for NpP. FM, considered a prototypical form of NpP, includes features of NeP, and its management is largely similar to that of NeP.
Hoegh (110)	NpP already serves as an evidence-based explanation that can help make sense of pain without any known pathology.	Existing concepts, namely central sensitization and unexplained cases of central NeP, can explain pain without any known pathology.

Abbreviations: NcP, nociceptive pain; NeP, neuropathic pain; NpP, nociplastic pain; FM, fibromyalgia.

This paper has highlighted the key challenges and conceptual challenges related to the concept of NpP. Medical theories that fail to address these challenges should be critically re-evaluated. If the concept of NpP is to be maintained, it will be necessary to address the concerns outlined in this proposal after its publication. It is first necessary to acknowledge the inconvenient truth of the coexistence of the unified NeP and the separate NeP (see Table 3), and then to propose a method that may help address it. The method presented in this paper represents one possible approach.

**Table 3:** Critical challenges and limitations of the concept of nociplastic pain.

1. The difference between NeP and NpP is the presence or absence of a lesion or disease that can explain the cause of pain; however, no diagnostic method exists at present that can reliably distinguish lesions causing pain from those not causing pain without relying on the patient's self-reported pain.
2. Each physician must determine whether a lesion or disease can explain the cause of pain.
3. There is no established method to accurately distinguish NeP from NpP. Additionally, no diagnostic method exists to identify NpP excluding NeP.
4. Pain of unknown cause can be explained not only by NpP but also by central sensitization and unexplained cases of central NeP.
5. FM is regarded as a typical NpP, but it often includes NeP. The treatment of FM largely overlaps with that of NeP.
6. At present, no disease exists that represents NpP alone without NeP.
7. Both “NeP with NpP” and “NeP without NpP” coexist; however, this coexistence is rarely recognized.
8. Because new studies cite previous papers, confusion regarding “NeP” has become increasingly pronounced over time.
9. Research on NpP is ongoing, but diverse diagnostic criteria have caused confusion. In one study, patients meeting the diagnostic criteria for FM were classified as NpP.
10. At present, no treatment exists that is specifically effective for NpP.
11. Based on the combinations of NpP, NeP, and NcP, pain is classified into seven categories, making diagnosis even more difficult. Distinguishing treatment strategies for each category is even more challenging.
12. NpP has been one of the factors contributing to the use of the term “psychogenic pain.”
13. Because FM is a prototypical form of NpP, non-specialist clinicians tend to perceive the diagnosis and treatment of FM as challenging and may be more likely to withdraw from providing care.
14. Despite all these problems, NpP has not demonstrated sufficient clinical utility to overcome them.

Abbreviations: NcP, nociceptive pain; NeP, neuropathic pain; NpP, nociplastic pain; FM, fibromyalgia.

## 8 Conclusions

From an etiological perspective, the IASP classifies pain into three categories: NcP, NeP, and NpP. In clinical practice, distinguishing between NpP and NeP can be particularly challenging. They share many clinical characteristics, including hypersensitivity to pain, spontaneous pain. Currently, no evidence-based diagnostic method has been established for NpP, as defined by the IASP. Questionnaires were developed before the IASP officially adopted NpP in 2017. Therefore, they cannot reliably distinguish between NpP and NeP. There is a previously unrecognized academic uncertainty arising from the simultaneous coexistence of the unified NeP and the separate NeP. There is currently no established guideline for either the pharmacological or the non-pharmacological treatment of NpP. The treatment of FM, a typical NpP, closely resembles that of NeP. The theoretical concept of NpP has generated substantial uncertainty not only in pain research but also in clinical practice, particularly regarding diagnosis and treatment.

To simplify diagnosis and treatment, resolve this scholarly uncertainty, and improve the care of patients with pain, a provisional plan is proposed to integrate NpP into NeP and adopt the term NeP, along with three preliminary proposals until an evidence-based diagnostic method for NpP has been established. It is hoped that these four proposals will serve as constructive contributions toward advancing both the conceptual understanding of pain and its treatment.
